# Supplementary material for: Undiscovered bird extinctions obscure the true magnitude of human-driven extinction waves
Source: Nat Commun. 2023 Dec 19;14:8116. doi: 10.1038/s41467-023-43445-2 (PMC10730700; doi:10.1038/s41467-023-43445-2)
Supplement: Supplementary file 1 — Supplementary Information [file 41467_2023_43445_MOESM1_ESM.pdf]

Undiscovered bird extinctions obscure the true magnitude of human-driven extinction waves

Supplementary Information

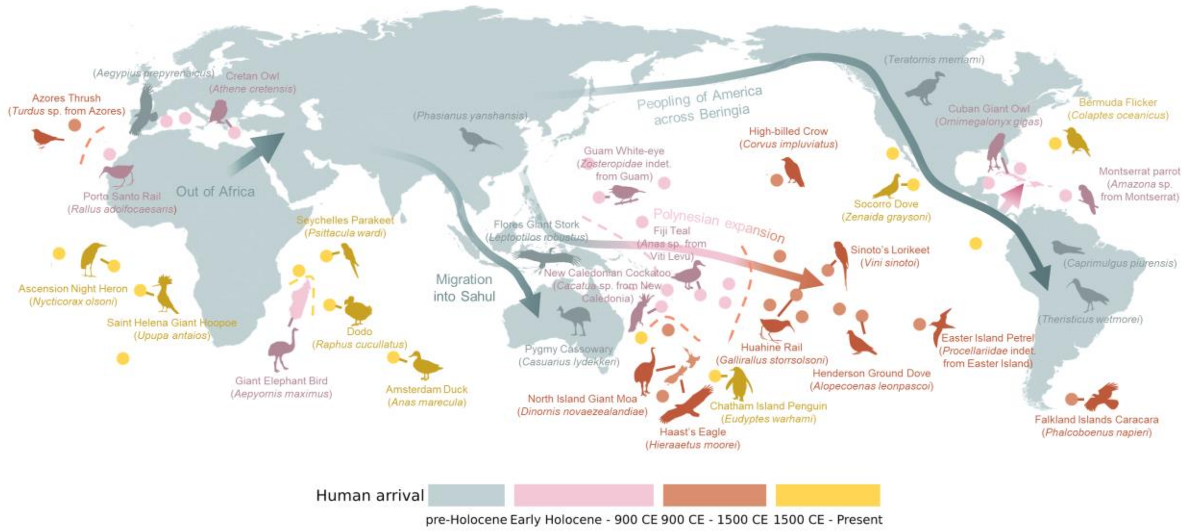

Supplementary Fig. 1: Human colonization of the globe, with additional detail. Human colonization is grouped into four major waves (see legend; pre-Holocene, Early Holocene - 900 CE, 900 CE - 1500 CE, 1500 CE - Present). Major human dispersal routes are indicated with arrows[1], with human migration into Eastern Polynesia estimated at 900 CE[2]. Regions are coloured based on their first human arrival; only regions with recorded (fossil or observed) bird extinctions are shown – see Supplementary Fig. 2 and Supplementary Data 1 for region locations and names. The map is centred on 145° E longitude. Icons show a range of example extinctions, with names. The icons are all from PhyloPic.org under Public Domain Dedication 1.0 licenses (see collection https://www.phylopic.org/collections/b2c5ed62-52af-0219-22e1-76a6538ce493). Creator credits: Birgit Lang (*Vini sinotoi* [*Psittacula derbiana*]), FJDegrange (*Colaptes oceanicus* [*Colaptes campestris*]), Ferran Sayol (*Amazona* sp. from

Montserrat [*Amazona aestiva*], *Athene cretensis* [*Athene noctua*], *Cacatua* sp. from New Caledonia [*Cacatua sulphurea*], *Caprimulgus piurensis* [*Caprimulgus*], *Casuarius lydekkeri* [*Casuarius casuarius*], *Leptoptilos robustus* [*Ciconia*], *Zenaida graysoni* [*Columba*], *Psittacula wardi* [*Cyanoliseus patagonus*], *Nycticorax olsoni* [*Nycticorax*], *Alopecoenas leonpascoi* [*Patagioenas*], *Rallus adolfocaesaris, Gallirallus storrsolsoni* [*Rallus*], *Upupa antaios* [*Upupa epops*], *Aegypius prepyrenaicus* [*Vultur gryphus*], *Zosteropidae* indet. from Guam [*Zosterops*]), Francesco "Architetto" Rollandin (*Turdus* sp. from Azores [*Turdidae*]), Juan Carlos Jerí (*Procellariidae* indet. from Easter Island [*Puffinus griseus*]), Mattia Menchetti (*Phasianus yanshansis* [*Phasianus colchicus*]), Peileppe (*Corvus impluviatus* [*Corvus brachyrhynchos*]), Rob Cooke (*Hieraaetus moorei* [*Accipitridae*], *Aepyornis maximus* [*Aepyornis maximus*], *Ornimegalonyx gigas* [*Ornimegalonyx gigas*], *Teratornis merriami* [*Teratornis merriami*], *Theristicus wetmorei* [*Theristicus wetmorei*]), Sean McCann (*Phalcoboenus napieri* [*Ibycter americanus*]), Sharon Wegner-Larsen (*Anas marecula*, *Anas* sp. from Viti Levu [*Anas fulvigula*]), and Steven Traver (*Eudyptes warhami* [*Aptenodytes patagonicus*], *Dinornis novaezealandiae* [*Dinornis novaezealandiae*], *Raphus cucullatus* [*Raphus cucullatus*]).

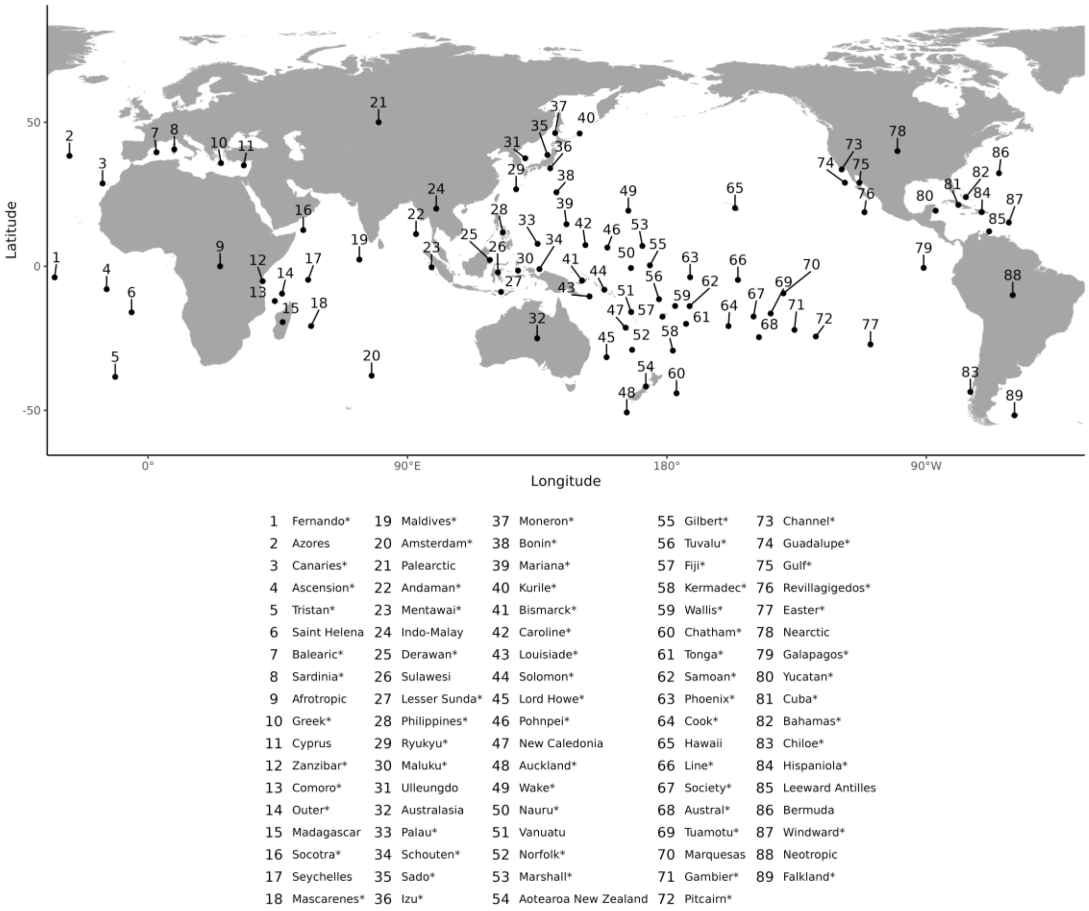

Supplementary Fig. 2: Global distribution of the 89 regions with bird extinctions. Includes all archipelagos and biogeographic realms[5] with bird extinctions. Archipelago names have been shortened for brevity where applicable – indicated with asterisks; for full archipelago names see Supplementary Data 1. The map is centred on 145° E longitude.

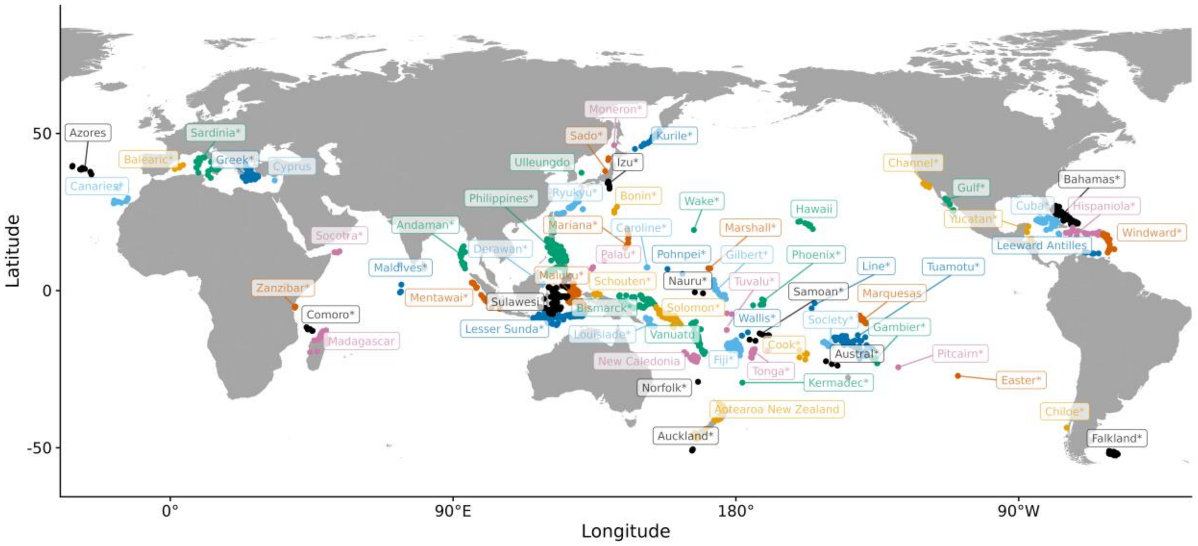

Supplementary Fig. 3: Archipelagos with the potential for undiscovered bird extinctions. We identified 1,488 islands (coloured areas/points) that were settled by humans pre-1500 CE, are >5 km^2, and were not covered by glaciers or connected to the mainland during the Last Glacial Maximum. We grouped these islands into 69 archipelagos. Aotearoa New Zealand is included for context, although we assume complete knowledge of the avifauna for Aotearoa New Zealand[3,4] and therefore zero undiscovered extinctions. Archipelago names have been shortened for brevity where applicable – indicated with asterisks; for full archipelago names see Supplementary Data 1. The map is centred on 145° E longitude.

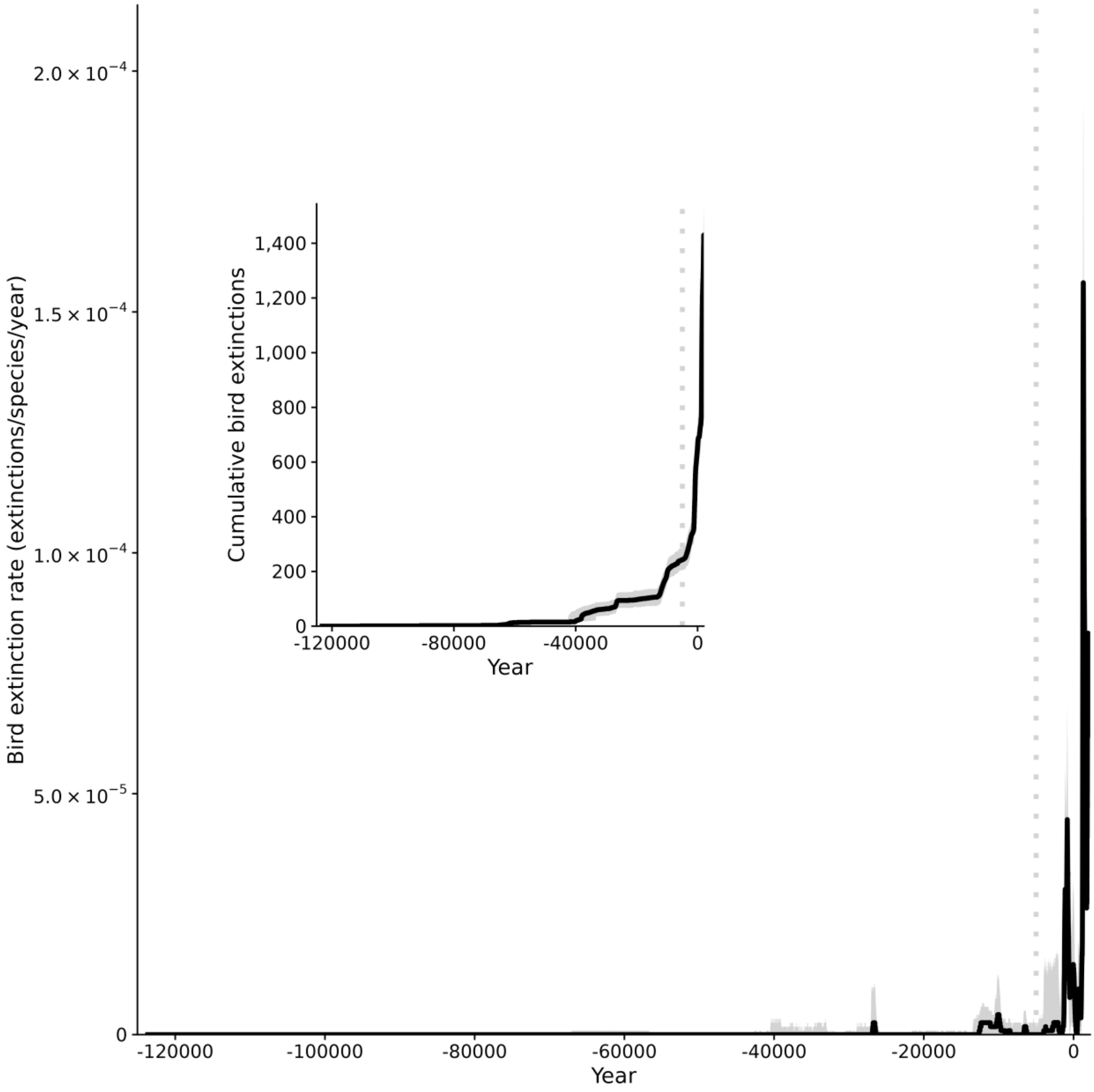

Supplementary Fig. 4: Bird extinction rate since the Late Pleistocene. Bird extinction rate is the median number of extinctions per species per year (extinctions/species/year) across 1,000 estimates of rolling means (100-year moving window; Supplementary Fig. 5); envelopes represent 95% credible intervals (n = 1,000). The inset shows cumulative bird extinctions since the Late Pleistocene. The dotted lines show the start of the focal period for Fig. 3 (5000 BCE). Source data are provided.

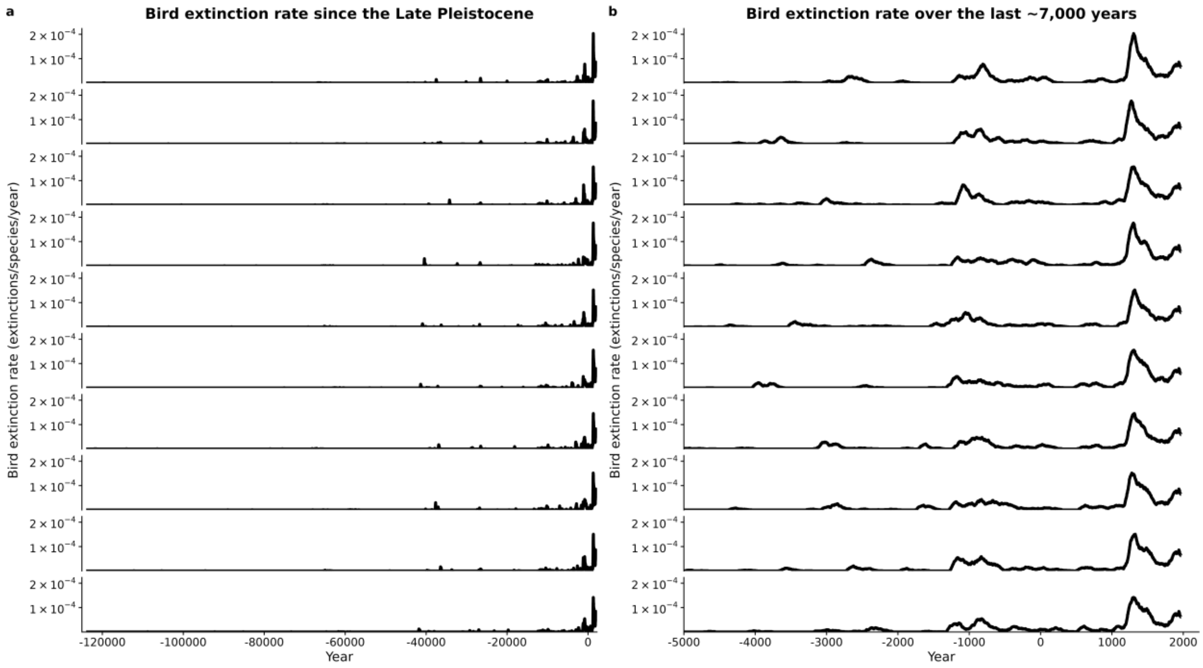

Supplementary Fig. 5: Individual estimates of bird extinction rate. a Bird extinction rate since the Late Pleistocene and **b** over the last ~7,000 years. Ten individual estimates out of 1,000 are displayed. Bird extinction rate is the number of extinctions per species per year (extinctions/species/year), averaged across a 100-year moving window for 10 separate estimates. The number of extinctions (variation in the number of undiscovered extinctions and possibly extinct species) and the timing of extinctions (uncertainty associated with human arrival dates and exponential decay – randomization of exponential extinction sequence) varies per estimate – see Methods. Source data are provided.

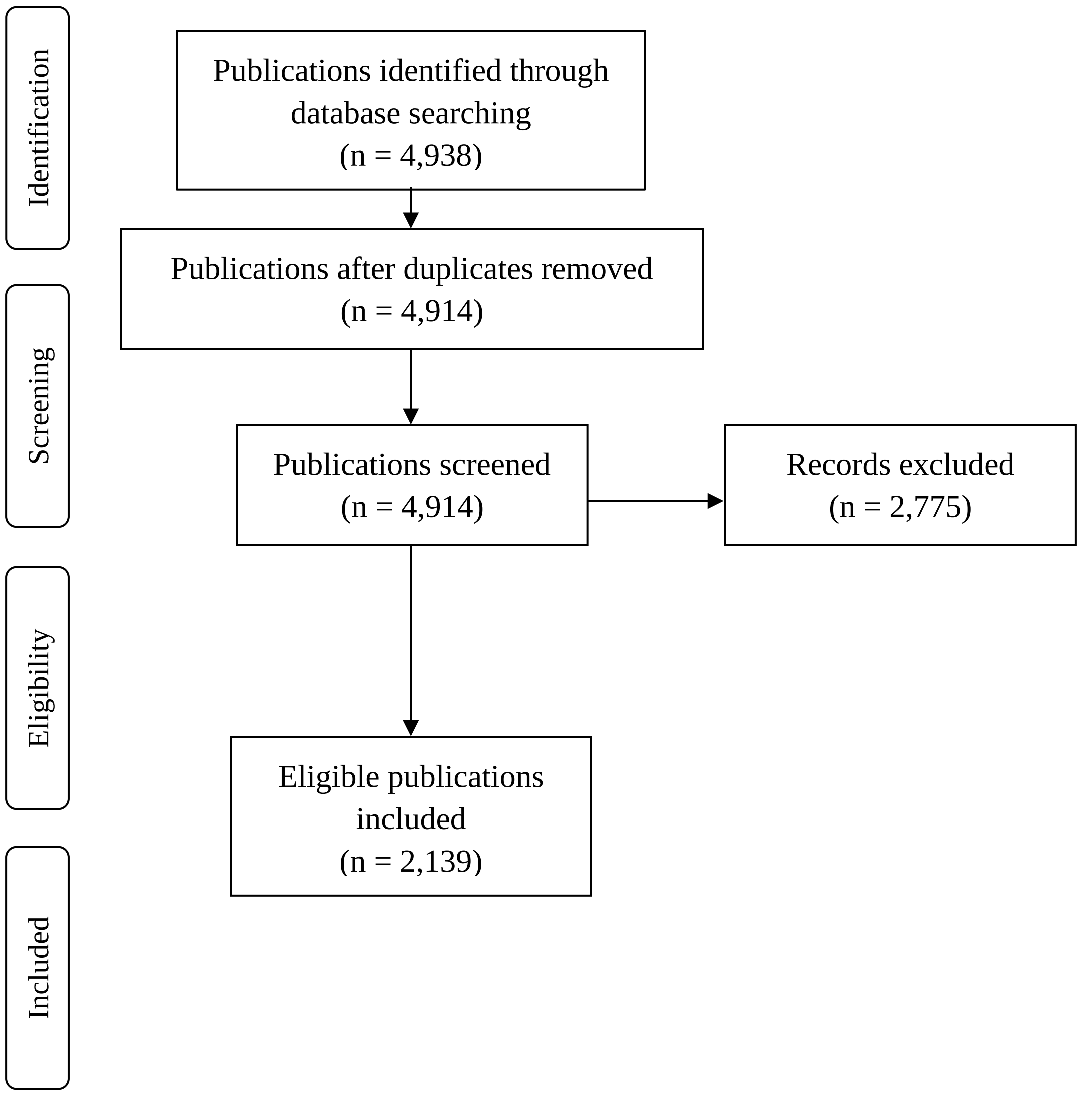

Supplementary Fig. 6: PRISMA flow diagram. Preferred Reporting Items for Systematic Reviews and Meta-Analysis (PRISMA)[6] flow diagram for research effort.

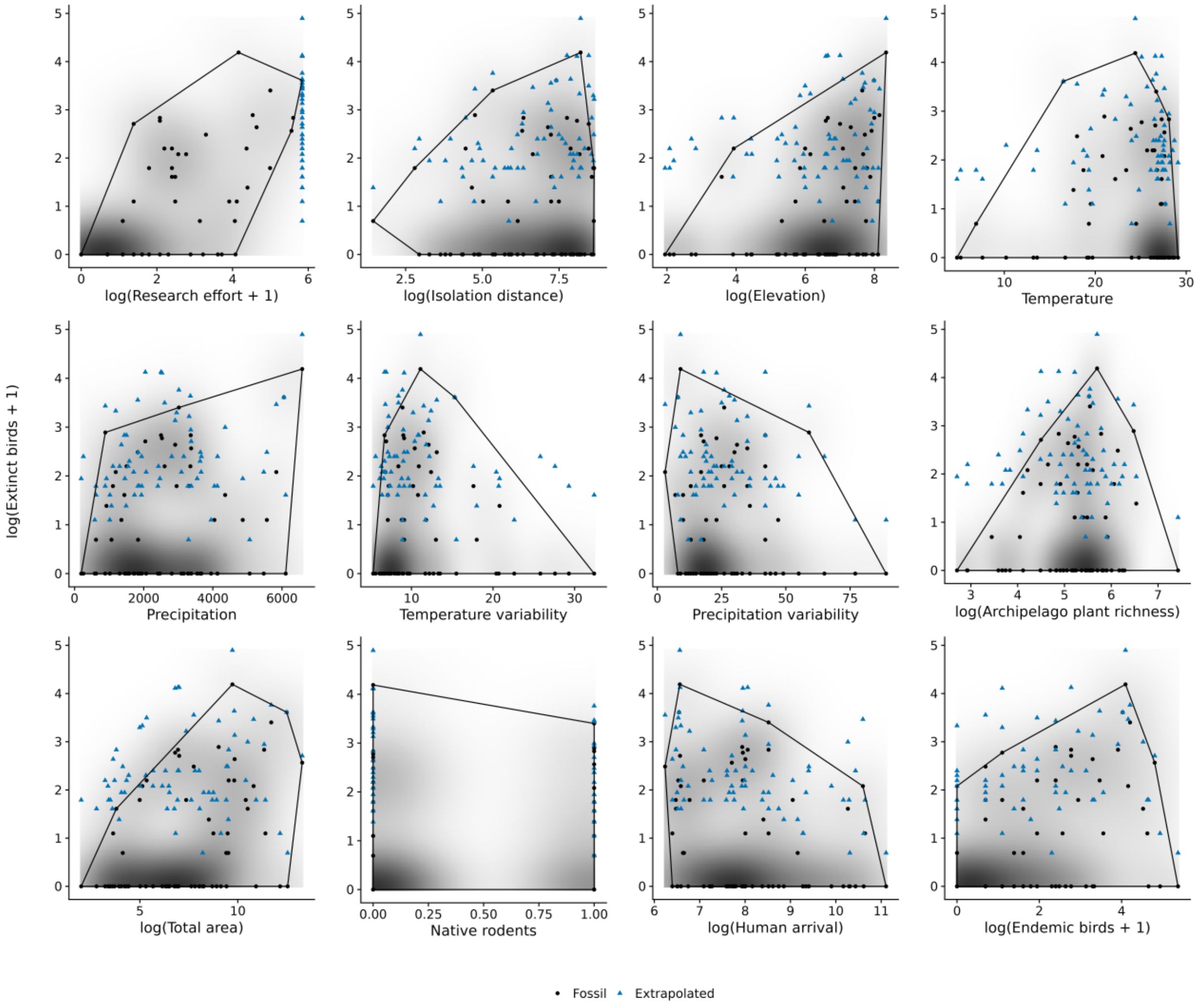

Supplementary Fig. 7: Comparison of fossil and extrapolated estimates of extinct birds. The fossil and extrapolated distributions of extinct birds for each predictor included in the model across 69 archipelagos (fossil n = 69, extrapolated n = 69). The extrapolated estimates are the median total of fossil + undiscovered (prior to rejection sampling) extinct birds per archipelago. Convex hulls (black lines) are drawn enclosing the recorded data (black dots). The colour gradient indicates regions of highest (black) to lowest (white) recorded data density. Extrapolated estimates that fall within the convex hulls can be recognized as interpolated, whereas extrapolated estimates outside the convex hulls can be recognized as extrapolated. Source data are provided.

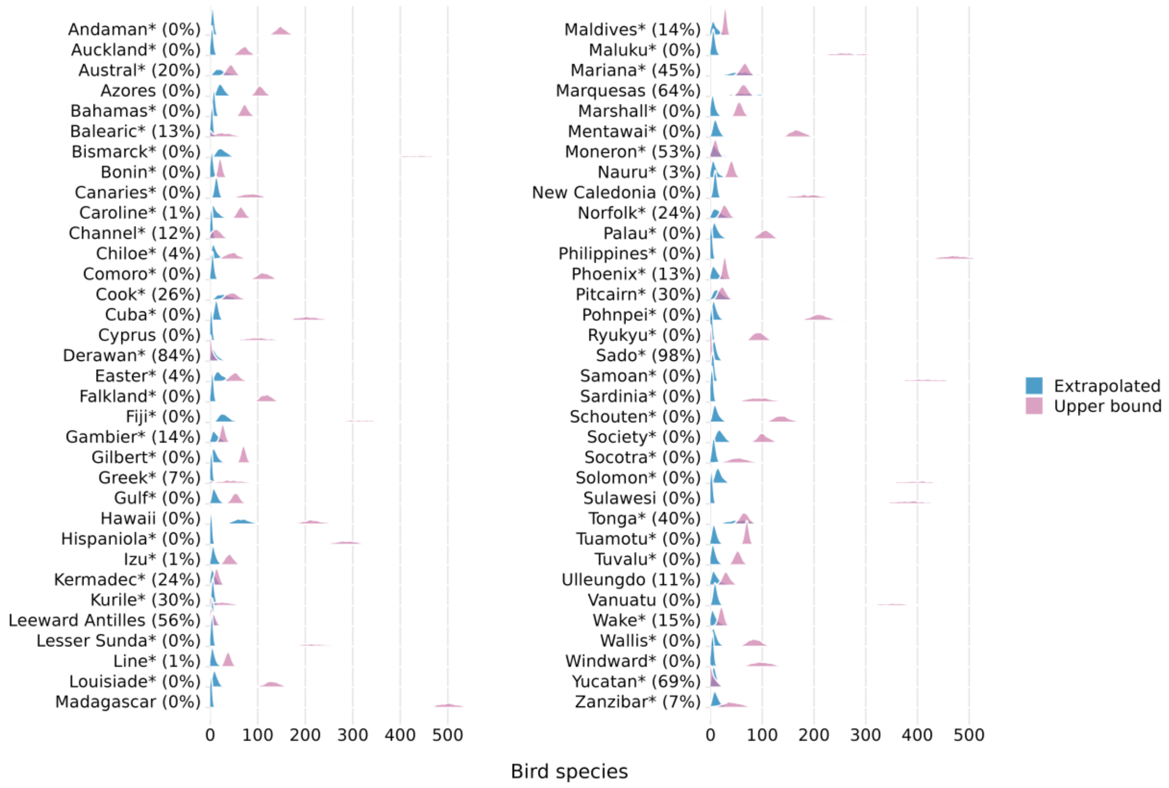

Supplementary Fig. 8: Archipelago upper bounds. Density distributions of extrapolated undiscovered extinct birds (extrapolations from the LM) and the upper bound of potential additional bird species (continental richness minus the number of fossil extinctions, observed extinctions, and extant bird species) across 68 archipelagos (excluding Aotearoa New Zealand) (extrapolated n = 1,000; upper bound n = 1,000 per archipelago). The numbers in brackets are the percent of records that were rejected during the rejection sampling. Source data are provided.

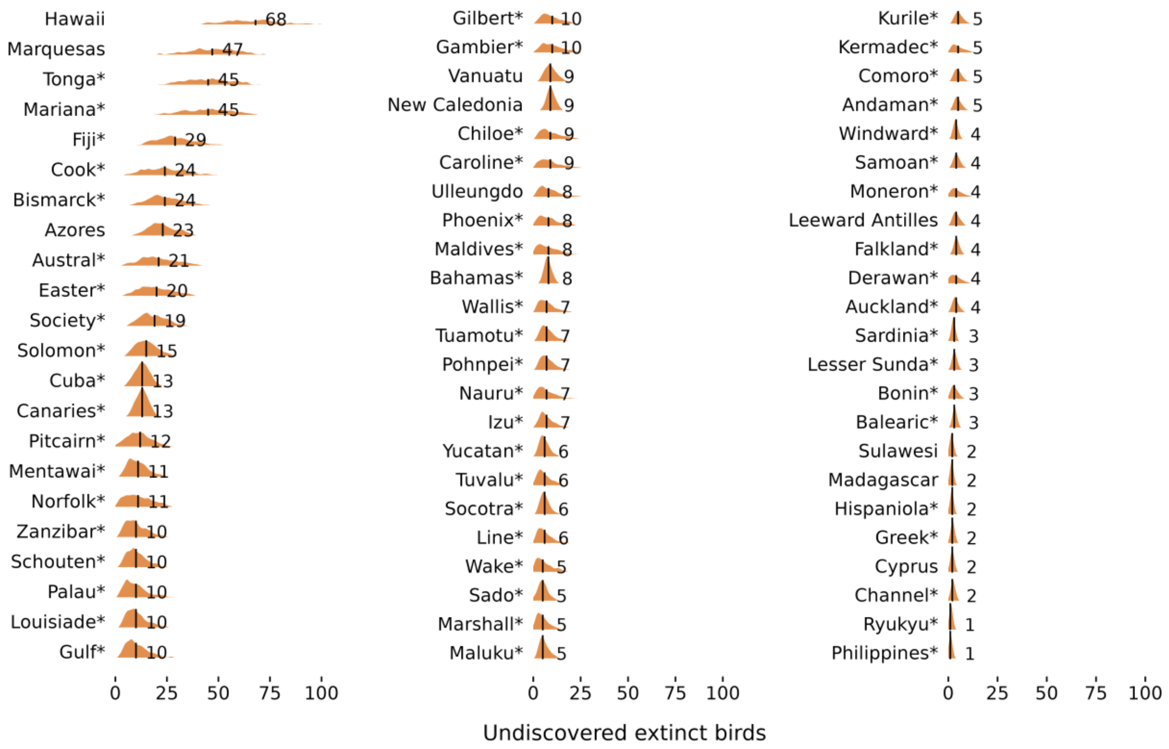

Supplementary Fig. 9: Distributions of undiscovered extinct birds across 68 archipelagos. Plots show the density distributions of undiscovered extinct birds per archipelago after rejection sampling, with a black line and label indicating the median estimate (n = 1,000 per archipelago). The 69 focal archipelagos minus Aotearoa New Zealand which is assumed to have zero undiscovered extinctions. Source data are provided.

Supplementary References

1. Nielsen, R. *et al.* Tracing the peopling of the world through genomics. *Nature* **541**, 302–310 (2017).
2. Sear, D. A. *et al.* Human settlement of East Polynesia earlier, incremental, and coincident with prolonged South Pacific drought. *Proceedings of the National Academy of Sciences of the United States of America* **117**, 8813–8819 (2020).
3. Holdaway, R. N., Worthy, T. H. & Tennyson, A. J. D. A working list of breeding bird species of the New Zealand region at first human contact. *New Zealand Journal of Zoology* **28**, 119–187 (2001).
4. Duncan, R. P., Boyer, A. G. & Blackburn, T. M. Magnitude and variation of prehistoric bird extinctions in the Pacific. *Proceedings of the National Academy of Sciences of the United States of America* **110**, 6436–6441 (2013).
5. Olson, D. M. *et al.* Terrestrial ecoregions of the worlds: a new map of life on Earth. *BioScience* **51**, 933–938 (2001).
6. Moher, D., Liberati, A., Tetzlaff, J. & Altman, D. G. Preferred reporting items for systematic reviews and meta-analyses: the PRISMA statement. *Annals of internal medicine* **151**, 264–269 (2009).
7. Chaubey, G. & Endicott, P. The Andaman islanders in a regional genetic context: reexamining the evidence for an early peopling of the archipelago from south Asia. *Human Biology* **85**, 153–172 (2013).
8. Wilmshurst, J. M., Hunt, T. L., Lipo, C. P. & Anderson, A. J. High-precision radiocarbon dating shows recent and rapid initial human colonization of East Polynesia. *Proceedings of the National Academy of Sciences of the United States of America* **108**, 1815–1820 (2011).
9. Hermann, A., Bollt, R. & Conte, E. The Atiahara site revisited: an early coastal settlement in Tubuai (Austral Islands, French Polynesia). *Archaeology in Oceania* **51**, 31–44 (2016).

10. Spínola, H., Brehm, A., Bettencourt, B., Middleton, D. & Bruges-Armas, J. HLA class I and II polymorphisms in Azores show different settlements in Oriental and Central islands. *Tissue Antigens* **66**, 217–230 (2005).

11. Berman, M. J. & Gnivecki, P. L. The Colonization of the Bahama Archipelago: A Reappraisal. *World Archaeology* **26**, 421–441 (1995).

12. Ramis, D., Alcover, J. A., Coll, J. & Trias, M. The Chronology of the First Settlement of the Balearic Islands. *Journal of Mediterranean Archaeology* **15**, 3–24 (2002).

13. Torrence, R. *et al.* Pleistocene Colonisation of the Bismarck Archipelago: New Evidence from West New Britain. *Archaeology in Oceania* **39**, 101–130 (2004).

14. Oda, S. The Archaeology of the Ogasawara Islands. *Asian Perspectives* **24**, 111–138 (1981).

15. Rando, J. C. *et al.* Phylogeographic patterns of mtDNA reflecting the colonization of the Canary Islands. *Annals of Human Genetics* **63**, 413–428 (1999).

16. Intoh, M. Human Dispersals into Micronesia. *AS* **105**, 15–28 (1997).

17. Kouvari, M. & van der Geer, A. A. E. Biogeography of extinction: The demise of insular mammals from the Late Pleistocene till today. *Palaeogeography, Palaeoclimatology, Palaeoecology* **505**, 295–304 (2018).

18. Álvarez Abel, R. Conchales Arqueológicos Y Comunidades Locales De Chiloé A Través De Una Experiencia De Educación Patrimonial. *Chungará (Arica)* **36**, 1151–1157 (2004).

19. Cheke, A. The timing of arrival of humans and their commensal animals on Western Indian Ocean oceanic islands. *Phelsuma* (2010).

20. Sondaar, P. & van der Geer, A. Mesolithic Environment and Animal Exploitation on Cyprus and Sardinia/Corsica. in *Archaeozoology of the Near East, ARC Publications* vol. 32 67–73 (2000).

21. Kusuma, P. *et al.* The last sea nomads of the Indonesian archipelago: genomic origins and dispersal. *Eur J Hum Genet* **25**, 1004–1010 (2017).

22. Hamley, K. M. *et al.* Evidence of prehistoric human activity in the Falkland Islands. *Science Advances* **7**, eabh3803 (2021).

23. Kumar, R. *et al.* The earliest-known humans in Fiji and their pottery: the first dates from the 2002 excavations at Naitabale (Naturuku), Moturiki Island. *S. Pac. J. Nat. App. Sci.* **22**, 16–22 (2004).

24. Thomas, F. R. Historical Ecology in Kiribati: Linking Past with Present. *Pacific Science* **63**, 567–600 (2009).

25. Des Lauriers, M. R. Terminal Pleistocene and Early Holocene Occupations of Isla de Cedros, Baja California, Mexico. *The Journal of Island and Coastal Archaeology* **1**, 255–270 (2006).

26. Higham, T. & Johnson, L. The Prehistoric Chronology of Raoul Island, the Kermadec Group. *Archaeology in Oceania* **32**, 207–213 (1997).

27. Tezuka, K. & Fitzhugh, B. New Evidence for Expansion of the Jomon Culture and the Ainu into the Kuril lslands: from IKIP 2000 Anthropological Research in the Kuril lslands. 北海道大学総合博物館研究報告 **2**, 85–95 (2004).

28. Shaw, B. & Dickinson, W. R. Excavation on Nimowa Island, Louisiade Archipelago, Papua New Guinea: Insights Into Cultural Practices and the Development of Exchange Networks in the Southern Massim Region. *The Journal of Island and Coastal Archaeology* **12**, 398–427 (2017).

29. Mohamed, N. Note on the Early History of the Maldives. *Archipel* **70**, 7–14 (2005).

30. O’Connor, S., Spriggs, M. & Veth, P. On the Cultural History of the Aru Islands: Some Conclusions. *The Archaeology of the Aru Islands* (2005).

31. Rieth, T. M. & Athens, J. S. Late Holocene Human Expansion into Near and Remote Oceania: A Bayesian Model of the Chronologies of the Mariana Islands and Bismarck Archipelago. *The Journal of Island and Coastal Archaeology* **14**, 5–16 (2019).

32. Weisler, M. I. Atolls as settlement landscapes: Ujae, Marshall Islands. *Atoll Research Bulletin* **460**, 1-51 (1999).

33. Forestier, H. *et al.* Le site de Tögi Ndrawa, île de Nias, Sumatra nord : les premières traces d'une occupation hoabinhienne en grotte en Indonésie. *Comptes Rendus Palevol* **4**, 727–733 (2005).

34. Kuzmin, Y. V. & Glascock, M. D. Two Islands in the Ocean: Prehistoric Obsidian Exchange between Sakhalin and Hokkaido, Northeast Asia. *The Journal of Island and Coastal Archaeology* **2**, 99–120 (2007).

35. Department of Economic Development and Environment. *First National Report To the United Nations Convention to Combat Desertification (UNCCD) Republic of Nauru*. https://library.sprep.org/content/first-national-report-united-nations-convention-combat-desertification-unccd-republic-nauru (2003).

36. Stevenson, J. & Dodson, J. R. Palaeoenvironmental Evidence for Human Settlement of New Caledonia. *Archaeology in Oceania* **30**, 36–41 (1995).

37. Anderson, A. & White, P. Prehistoric settlement on Norfolk Island and its Oceanic context. *Rec. Aust. Mus., Suppl.* **27**, 135–141 (2001).

38. Fitzpatrick, S. M. & Jew, N. P. Radiocarbon dating and Bayesian modelling of one of Remote Oceania's oldest cemeteries at Chelechol ra Orrak, Palau. *Antiquity* **92**, 149–164 (2018).

39. Mijares, A. S. *et al.* New evidence for a 67,000-year-old human presence at Callao Cave, Luzon, Philippines. *Journal of Human Evolution* **59**, 123–132 (2010).

40. Carson, M. T. Cultural Affinities of Monumental Architecture in the Phoenix Islands. *The Journal of the Polynesian Society* **107**, 61–77 (1998).

41. Steadman, D. W. & Olson, S. L. Bird remains from an archaeological site on Henderson Island, South Pacific: Man-caused extinctions on an "uninhabited" island. *Proceedings of the National Academy of Sciences* **82**, 6191–6195 (1985).

42. Rainbird, P. Kosrae's place in Pacific prehistory. *Archaeology in Oceania* **30**, 139–145 (1995).

43. Watanobe, T. *et al.* Prehistoric Sado Island Populations of Sus scrofa Distinguished from Contemporary Japanese Wild Boar by Ancient Mitochondrial DNA. *jzoo* **21**, 219–228 (2004).

44. Anderson, A. The rat and the octopus: initial human colonization and the prehistoric introduction of domestic animals to Remote Oceania. *Biol Invasions* **11**, 1503–1519 (2009).

45. Cerný, V. *et al.* Out of Arabia-the settlement of island Soqotra as revealed by mitochondrial and Y chromosome genetic diversity. *Am J Phys Anthropol* **138**, 439–447 (2009).

46. Wickler, S. & Spriggs, M. Pleistocene human occupation of the Solomon Islands, Melanesia. *Antiquity* **62**, 703–706 (1988).

47. Aubert, M. *et al.* Pleistocene cave art from Sulawesi, Indonesia. *Nature* **514**, 223–227 (2014).

48. Burley, D., Weisler, M. I. & Zhao, J. High Precision U/Th Dating of First Polynesian Settlement. *PLOS ONE* **7**, e48769 (2012).

49. Lilley, I., Allen, M., Clark, G. & Irwin, G. *Early human expansion and innovation in the Pacific: Thematic Study*. (ICOMOS, 2010).

50. Mills, S. & Park, S.-H. A Mysterious Island in the Digital Age: Technology and Musical Life on Ulleungdo, South Korea. *Ethnomusicology Forum* **22**, 160–187 (2013).

51. Bedford, S. & Spriggs, M. The Archaeology of Vanuatu: 3,000 Years of History across Islands of Ash and Coral. in *The Oxford Handbook of Prehistoric Oceania* (eds. Cochrane, E. E. & Hunt, T. L.) (Oxford University Press, 2018). doi:10.1093/oxfordhb/9780199925070.013.015.

52. Olson, S. L. & Rauzon, M. J. The Extinct Wake Island Rail Gallirallus wakensis: A Comprehensive Species Account Based on Museum Specimens and Archival Records. *wils* **123**, 663–689 (2011).

53. Sand, C. A View from the West: Samoa in the Culture History of `Uvea (Wallis) and Futuna (Western Polynesia). *Journal of Samoan Studies* **2**, 5-15 (2006).

54. Gompper, M. E., Petrites, A. E. & Lyman, R. L. Cozumel Island fox (Urocyon sp.) dwarfism and possible divergence history based on subfossil bones. *Journal of Zoology* **270**, 72–77 (2006).

55. Skoglund, P. *et al.* Reconstructing Prehistoric African Population Structure. *Cell* **171**, 59-71.e21 (2017).

56. Weigelt, P., Jetz, W. & Kreft, H. Bioclimatic and physical characterization of the world's islands. *Proceedings of the National Academy of Sciences of the United States of America* **110**, 15307–15312 (2013).

57. Weigelt, P. & Kreft, H. Quantifying island isolation - insights from global patterns of insular plant species richness. *Ecography* **36**, 417–429 (2013).

58. Hijmans, R. J., Cameron, S. E., Parra, J. L., Jones, P. G. & Jarvis, A. Very high resolution interpolated climate surfaces for global land areas. *International Journal of Climatology* **25**, 1965–1978 (2005).

59. Faurby, S. *et al.* PHYLACINE 1.2: the phylogenetic atlas of mammal macroecology. *Ecology* **99**, 2626 (2018).

60. Farr, T. G. *et al.* The shuttle radar topography mission. *Reviews of Geophysics* **45**, 1–33 (2007).
