# Supplementary material for: Undiscovered bird extinctions obscure the true magnitude of human-driven extinction waves
Source: Nat Commun. 2023 Dec 19;14:8116. doi: 10.1038/s41467-023-43445-2 (PMC10730700; doi:10.1038/s41467-023-43445-2)
Supplement: Supplementary file 3 — Description of Additional Supplementary Files [file 41467_2023_43445_MOESM3_ESM.pdf]

Description of Additional Supplementary Files

Supplementary Data file, including Supplementary Data 1-6:

Supplementary Data 1: Region abbreviations and full names. Ordered West-East (centred on 145° E longitude, for region numbers and locations see Supplementary Fig. 2. Region number is the number used in Supplementary Fig. 2; Region abbreviation is the abbreviation used for the region in the text and in Supplementary Fig. 3; Region full name describes the region in more detail.

Supplementary Data 2: Date of first human arrival for the 69 focal archipelagos. Archipelago is the (abbreviated) archipelago name; Archipelago full name is the full name for the archipelago; Best is the best estimate of first human arrival for the archipelago from the literature; Lower is the lower estimate of first human arrival where a range is provided in the literature; Upper is the upper estimate of first human arrival where a range is provided in the literature; Reference is the source of the estimate of first human arrival, with the number reflecting that referred to in the Supplementary Information; Full reference is the full reference for the source of the estimate.

Supplementary Data 3: Predictors of fossil extinct birds. Predictor lists potential predictors of the number of fossil extinct bird species per archipelago; Description provides definitions for the predictor; Included specifies whether the predictor was included in the model(s) of fossil bird extinctions; Aggregation details how the island estimates, where applicable, were aggregated to the archipelago scale; Transformation details if/how the predictors were transformed prior to modelling; Reference lists the sources of data for the predictors, with the number reflecting that referred to in the Supplementary Information; Full reference is the full reference for the data source.

Supplementary Data 4: Coefficients for the simple linear model of fossil extinct birds. Predictors were centred and scaled to improve the interpretability of the regression coefficients. Predictors details the predictors included in the simple linear model of fossil extinct birds; Coefficient is the resulting coefficients from the simple linear model; Standard error is the standard error associated with these coefficients; Partial R2 is the partial R^2 associated with the predictor.

Supplementary Data 5: Global environmental predictors per grid cell used to model the upper bound. Predictor lists predictors of maximum potential bird diversity included in the upper bound model; Description provides definitions of the predictors; Transformation details if/how the predictors were transformed prior to modelling; Reference lists the sources of data for the predictors, with the number reflecting that referred to in the Supplementary Information; Full reference is the full reference for the data source.

Supplementary Data 6: Coefficients for the upper bound model. Predictors were centred and scaled to improve the interpretability of the regression coefficients. Predictor lists predictors of maximum potential bird diversity included in the upper bound model; Coefficient is the resulting coefficients from the upper bound model after including the residuals autocovariate term (Spatial-model) and before including the residuals autocovariate term (Non-spatial model); Standard error is the standard error associated with the coefficients after including the residuals autocovariate term (Spatial-model) and before including the residuals autocovariate term (Non-spatial model).

Source Data file: source data for Figures 2, 3, 5, and Supplementary Figures 4, 5, 7, 8 and 9.
