# Supplementary material for: Undiscovered bird extinctions obscure the true magnitude of human-driven extinction waves
Source: Nat Commun. 2023 Dec 19;14:8116. doi: 10.1038/s41467-023-43445-2 (PMC10730700; doi:10.1038/s41467-023-43445-2)
Supplement: Supplementary file 5 — Reporting Summary [file 41467_2023_43445_MOESM5_ESM.pdf]

nature portfolio

Corresponding author(s): Rob Cooke

Last updated by author(s): Oct 16, 2023

Reporting Summary

Nature Portfolio wishes to improve the reproducibility of the work that we publish. This form provides structure for consistency and transparency in reporting. For further information on Nature Portfolio policies, see our Editorial Policies and the Editorial Policy Checklist.

Statistics

For all statistical analyses, confirm that the following items are present in the figure legend, table legend, main text, or Methods section.

n/a	Confirmed	
☐	☒	The exact sample size (*n*) for each experimental group/condition, given as a discrete number and unit of measurement
☒	☐	A statement on whether measurements were taken from distinct samples or whether the same sample was measured repeatedly
☐	☒	The statistical test(s) used AND whether they are one- or two-sided<br>*Only common tests should be described solely by name; describe more complex techniques in the Methods section.*
☐	☒	A description of all covariates tested
☐	☒	A description of any assumptions or corrections, such as tests of normality and adjustment for multiple comparisons
☐	☒	A full description of the statistical parameters including central tendency (e.g. means) or other basic estimates (e.g. regression coefficient) AND variation (e.g. standard deviation) or associated estimates of uncertainty (e.g. confidence intervals)
☐	☒	For null hypothesis testing, the test statistic (e.g. *F*, *t*, *r*) with confidence intervals, effect sizes, degrees of freedom and *P* value noted<br>*Give P values as exact values whenever suitable.*
☐	☒	For Bayesian analysis, information on the choice of priors and Markov chain Monte Carlo settings
☒	☐	For hierarchical and complex designs, identification of the appropriate level for tests and full reporting of outcomes
☐	☒	Estimates of effect sizes (e.g. Cohen's *d*, Pearson's *r*), indicating how they were calculated

Our web collection on statistics for biologists contains articles on many of the points above.

Software and code

Policy information about availability of computer code

Data collection	No software was used for data collection.
Data analysis	The primary code for data analysis was written in R using versions up to 4.0.4. The code was tested for compatibility on R version 4.0.4. The code written to perform the data analyzes is available at: https://zenodo.org/records/10014585. All R packages used to perform the analyzes are listed within the code and are referenced in the main text. The list of packages used is: arm 1.13-1, broom 1.0.4, cowplot 1.1.1, DHARMa 0.4.6, dplyr 1.1.2, gghalves 0.1.4, ggplot2 3.4.2, HDInterval 0.2.4, hydroGOF 0.4-0, jtools 2.2.1, letsR 4.0, MASS 7.3-53, purrr 1.0.1, raster 3.6-20, readr 2.1.4, rsq 2.5, scales 1.2.1, scatterpie 0.1.8, sf 1.0-13, sp 1.6-0, tibble 3.2.1, tidyr 1.3.0, and zoo 1.8-12. See the renv for details of the dependencies. In addition, preprocessing of shapefiles of the archipelagos was performed in ArcGIS 10.4 and rasterization of landmasses was run in Python version 2.7.10.

For manuscripts utilizing custom algorithms or software that are central to the research but not yet described in published literature, software must be made available to editors and reviewers. We strongly encourage code deposition in a community repository (e.g. GitHub). See the Nature Portfolio guidelines for submitting code & software for further information.

Data

Policy information about availability of data

All manuscripts must include a data availability statement. This statement should provide the following information, where applicable:

- Accession codes, unique identifiers, or web links for publicly available datasets
- A description of any restrictions on data availability
- For clinical datasets or third party data, please ensure that the statement adheres to our policy

All prepared and processed data are available at <https://zenodo.org/records/10014585> (doi: 10.5281/zenodo.10014585). Raw data on island characteristics (e.g., island area, island precipitation)66 are available from <https://doi.org/10.5061/dryad.fv94v>. Shapefiles of the archipelagos are available from the database of global administrative areas (GADM) <gadm.org/data.html>. Information on fossil extinct birds72 is available from <https://doi.org/10.5061/dryad.s1rn8pk66>. Raw information on native rodents97 is available from <https://doi.org/10.5281/zenodo.1250504>. Shapefiles of bird distributions are available from <http://datazone.birdlife.org/species/requestdis>. Global elevation data80 are available from <https://srtm.csi.cgiar.org/srtmdata/>, while WorldClim79 data are available from <https://www.worldclim.org/data/worldclim21.html> or via the getData function from the raster R package55. Bird extinction probabilities and the date of recent bird extinctions22 are available from <https://data.mendeley.com/datasets/vvjhpmyxb4/1>. Source data are also provided with this paper. Specifically, the source data underlying Figs. 2, 3 and 5, and Supplementary Figs. 4, 5, 7, 8 and 9 are provided.

Research involving human participants, their data, or biological material

Policy information about studies with human participants or human data. See also policy information about sex, gender (identity/presentation), and sexual orientation and race, ethnicity and racism.

Reporting on sex and gender	NA
Reporting on race, ethnicity, or other socially relevant groupings	NA
Population characteristics	NA
Recruitment	NA
Ethics oversight	NA

Note that full information on the approval of the study protocol must also be provided in the manuscript.

Field-specific reporting

Please select the one below that is the best fit for your research. If you are not sure, read the appropriate sections before making your selection.

☐ Life sciences ☐ Behavioural & social sciences ☒ Ecological, evolutionary & environmental sciences

For a reference copy of the document with all sections, see nature.com/documents/nr-reporting-summary-flat.pdf

Ecological, evolutionary & environmental sciences study design

All studies must disclose on these points even when the disclosure is negative.

Study description	We compiled existing data to model and estimate the number and timing of bird extinctions since the Late Pleistocene across the globe. No primary investigation was undertaken, instead we extracted/collated existing data from multiple sources detailed in the manuscript.
Research sample	We obtained information for all known observed and fossil bird extinctions since the Late Pleistocene (the last ~126,000 years) until the present day (2018). The sample therefore reflects all global bird extinctions during the period of dominance of modern humans. Specifically, we extracted all recorded fossil bird extinctions from Sayol et al. 2020 <https://doi.org/10.5061/dryad.s1rn8pk66> and all known observed extinctions from Butchart et al. 2018 <https://data.mendeley.com/datasets/vvjhpmyxb4/1> cross-checked against the IUCN Red List. This dataset covers all known bird extinctions globally (616 species).
Sampling strategy	All known bird extinctions have been included, so no sampling was performed. No sample-size calculations were performed beforehand; we used all of the data that was available. Sample size = population size.
Data collection	Data were compiled from multiple sources and are cited in the manuscript. Rob Cooke downloaded the relevant datasets. The data included geographic and environmental data for 17,883 islands <https://doi.org/10.5061/dryad.fv94v>, human settlement information for the focal archipelagos from the literature (Supplementary Data 2), data on fossil extinct birds (pre-1500 CE) <https://doi.org/10.5061/dryad.s1rn8pk66>, data on observed bird extinctions (post-1500 CE) <https://data.mendeley.com/datasets/vvjhpmyxb4/1>, BirdLife range maps <http://datazone.birdlife.org/species/requestdis>, shapefiles of the archipelagos <gadm.org/

	data.html>, information on native rodents <https://doi.org/10.5281/zenodo.1250504>, global elevation data <https://srtm.csi.cgiar.org/srtmdata/>, and global climate data <https://www.worldclim.org/data/worldclim21.html>.
Timing and spatial scale	Data represent the globe for the period between the Late Pleistocene (the last ~126,000 years) and the present day (2018). Data were compiled between 2020 and 2021. Specifically, fossil bird extinctions were obtained from a publication published in 2020 <https://doi.org/10.5061/dryad.s1rn8pk66>, BirdLife range maps from BirdLife version 2018.1, research effort was calculated from a search performed in 2020, and information on possibly extinct species was obtained from a publication published in 2018 <https://data.mendeley.com/datasets/vvjhpmyxb4/1>.
Data exclusions	No data were excluded from the analysis.
Reproducibility	No experiments were conducted for this study. Analytical reproducibility is underpinned by the R notebook available at <https://zenodo.org/records/10014585>
Randomization	No experiments were conducted for this study.
Blinding	No experiments were conducted for this study. Data were collated from previously published literature and blinding was not relevant.

Did the study involve field work? ☐ Yes ☒ No

Reporting for specific materials, systems and methods

We require information from authors about some types of materials, experimental systems and methods used in many studies. Here, indicate whether each material, system or method listed is relevant to your study. If you are not sure if a list item applies to your research, read the appropriate section before selecting a response.

Materials & experimental systems

n/a	Involved in the study	
☒	☐	Antibodies
☒	☐	Eukaryotic cell lines
☒	☐	Palaeontology and archaeology
☒	☐	Animals and other organisms
☒	☐	Clinical data
☒	☐	Dual use research of concern
☒	☐	Plants

Methods

n/a	Involved in the study	
☒	☐	ChIP-seq
☒	☐	Flow cytometry
☒	☐	MRI-based neuroimaging
